# Healthcare professionals’ trust in health authorities throughout COVID-19: a social media analysis

**DOI:** 10.1038/s41598-026-50565-4

**Published:** 2026-04-30

**Authors:** Idan-Chaim Cohen, Noa Tal, Aviad Elyashar, Rami Puzis, Odeya Cohen

**Affiliations:** 1 Department of Nursing, Recanati School of Community Health Professions, Faculty of Health Sciences, Ben-Gurion University of the Negev, Be’er Sheva, Israel; 2 Cyber@BGU, Ben-Gurion University of the Negev, Be’er Sheva, Israel; 3 The Stein Faculty of Computer and Information Science, Ben-Gurion University of the Negev, Be’er Sheva, Israel; 4 Department of Computer Science, Shamoon College of Engineering, Be’er Sheva, Israel

**Keywords:** Healthcare professionals, Institutional trust, COVID-19, Social media analysis, Large language models, Health authorities, Diseases, Health care, Medical research

## Abstract

Healthcare professionals’ institutional trust in health authorities is important for effective crisis response, as these professionals serve as both implementers of health directives and trust intermediaries influencing public confidence. Despite extensive research on public trust during COVID-19, studies examining healthcare professionals’ trust in health authorities remain limited. Traditional survey methods face declining response rates and provide only cross-sectional snapshots. Social media platforms offer longitudinal data enabling analysis of trust expressions over extended periods. We analyzed 50,308 tweets from 9,442 healthcare professionals mentioning WHO, CDC, and FDA from January 2019 through March 2023. Large language models assessed trust levels using a 5-point scale. WHO showed the highest mean trust score, followed by FDA and CDC. Trust patterns varied by authority: WHO showed a pronounced early-pandemic decline with subsequent recovery, CDC showed sustained trust erosion throughout 2022–2023, and FDA remained relatively stable. Topic modeling identified 10 major themes, with integrity concerns dominating low-trust discourse across all authorities. Social media analysis demonstrates feasibility for continuous monitoring of healthcare professionals’ trust dynamics, enabling early detection of authority-specific trust erosion patterns.

## Introduction

Crisis response requires trust among stakeholders to enable effective collaboration^1–3^. Institutional trust that healthcare professionals (HCPs) place in health authorities (HAs) is particularly critical. HAs examined in this study include the World Health Organization (WHO), the United States Centers for Disease Control and Prevention (CDC), and the United States Food and Drug Administration (FDA). Institutional trust refers to individuals’ belief that a public institution has the will and ability to act according to their values^4^. The OECD framework identifies two drivers of institutional trust^5^: values (ethical standards and fairness demonstrated by institutions) and competencies (institutional capacity to design and deliver policies effectively).

HCPs’ institutional trust in HAs influences health outcomes through two mechanisms. First, HAs develop and disseminate health guidelines to the public and HCPs, with HCPs serving as implementation agents. HCPs’ trust in HAs correlates with adherence to HA directives during health crises, particularly pandemics^6–8^. This adherence is associated with implementation effectiveness and health outcomes^9–11^. Second, HCPs’ trust in HAs may influence public trust through transitive mechanisms^12^, where the public may use HCPs’ expressed trust to inform their own assessments of HA credibility. When HCPs express trust or distrust in HAs through communications and practices, the public may incorporate these signals into their own trust evaluations^13^. HCPs’ institutional trust in HAs thus affects both direct implementation of health directives and indirect influence on public trust.

COVID-19 led to widespread declines in institutional trust^14^. A longitudinal study of 806 US respondents found institutional trust in HAs declined substantially over four months (March–July 2020), with trust in the CDC decreasing by 28% and trust in US state health departments decreasing by 24.2%^15^. These declines may have stemmed from perceptions of political influence on health recommendations, inconsistent messaging, and apparent deviations from evidence-based approaches^16^. COVID-19 research revealed institutional trust’s dynamic nature and vulnerability to rapid change during prolonged health emergencies^17^. Given HCPs’ dual role as implementers of HA directives and public trust influencers, understanding HCPs’ trust dynamics is particularly important.

While institutional trust among the general public during health crises has been well studied, research specifically examining HCPs’ trust in HAs remains limited. Additionally, existing health trust research predominantly relies on cross-sectional designs, presenting methodological constraints. First, empirical trust research is typically cross-sectional; however, this design only provides snapshots at specific time points, which may not fully capture trust as a dynamic phenomenon^18^. Second, trust measurements commonly rely on survey methodologies, but response rates have declined over recent decades^19,20^. This trend may compromise data representativeness, though this limitation’s extent varies across study populations and contexts.

Social media research may help address these methodological limitations. Twitter serves as a platform where HCPs create and share professional content^21^, providing longitudinal data that enables systematic analysis of authentic communication over extended periods^22^. Social media analysis may address survey limitations by providing continuous temporal data and reducing reliance on voluntary survey participation.

This study analyzes trust dynamics expressed by HCPs on Twitter toward WHO, CDC, and FDA from January 2019 to March 2023. Specifically, we aim to: (1) track trust level fluctuations over time; (2) identify topics associated with high versus low trust expressions; and (3) compare trust patterns across the three HAs.

## Methods

### Reporting standards

This observational study was conducted and reported according to the Strengthening the Reporting of Observational Studies in Epidemiology (STROBE) guidelines^23^. The completed STROBE checklist is provided as Supplementary Note.

### Study design

This longitudinal observational study analyzed publicly available Twitter discourse from healthcare professionals regarding WHO, CDC, and FDA between January 2019 and March 2023. Trust levels were assessed using large language model analysis of individual tweets. Topic modeling identified discourse themes associated with high versus low trust expressions.

### Ethical considerations

The collection, storage, and analysis methods for the Twitter panel were approved by the Institutional Review Board (IRB) of Ben-Gurion University of the Negev (Approval #1879-1, and SISE-2025-50). All methods were performed in accordance with the relevant guidelines and regulations. The IRB of Ben-Gurion University of the Negev determined that individual informed consent was not required, as the study involved analysis of publicly available, self-disclosed data with no direct participant interaction. Author identifiers were excluded from all analyses and will not be shared. All data were stored on a secured server. Tweet texts were accessible only to the research team, who maintained data confidentiality. Non-author members of the research team who validated LLM classifications using publicly available tweets signed confidentiality agreements and retained no data. To prevent identification of tweet authors through text searches, tweet content is not included in shared datasets. The Twitter dataset^24^ was used with permission from the dataset owners.

### Dataset acquisition and filtering

We used the dataset created by Elyashar et al.^24^, containing over 28 million English tweets from January 2019 to March 2023 authored by more than 53,000 HCPs. HCPs were defined as individuals working in the healthcare system or studying a medical profession, including physicians, nurses, pharmacists, and medical students, while excluding complementary and alternative medicine practitioners and organizational accounts. HCPs were identified using supervised classification (support vector machines) trained on self-reported biographical text in Twitter profiles, achieving 90% accuracy on manual verification of 100 randomly sampled accounts. Using regular expressions, we identified tweets mentioning FDA, CDC, and WHO by searching for terms listed in Supplementary Table S1. This yielded 50,308 tweets from 9,442 HCPs, distributed as follows: WHO (19,801), CDC (18,461), and FDA (12,046). The Twitter dataset did not include sex or gender information.

### Tweet volume analysis

We segmented the dataset into quarterly periods (January–March, April–June, July–September, and October–December), resulting in 17 consecutive periods from Q1 2019 through Q1 2023. Tweet volumes were counted for each authority per quarter. Visual analysis was used to identify temporal patterns and peaks. Fold changes between consecutive quarters were calculated.

### Trust score analysis

Deepseek-r1, a 7.62B-parameter, reasoning-optimized LLM^25^, was used to evaluate trust expressed in tweets. Each tweet was analyzed using a standardized prompt instructing the model to assess author’s trust toward the mentioned HA using a 5-point Likert-type scale: 1 (no trust), 2 (low), 3 (neutral/unclear), 4 (high), and 5 (complete). A zero-shot approach (without examples) was used to minimize example-selection bias and reduce processing complexity. The scale used descriptive labels (no trust, low trust, neutral or unclear, high trust, complete trust) without detailed definitions for each level. This approach is consistent with OECD trust measurement guidelines, which recommend that respondents draw on their own understanding of trust rather than being provided with formal definitions^4^. Human raters received the same instructions and scale as the LLM. Illustrative examples for each trust level are provided in Supplementary Table S6. The full prompt is provided in Supplementary Methods.

### Validation of trust score analysis

To validate the LLM-based trust assessment and address potential measurement bias, three independent reviewers rated a stratified random sample of 300 tweets (100 per authority), with proportional representation of trust categories (low 1–2, neutral 3, high 4–5) within each authority and a minimum of 20 tweets per stratum. Reviewers followed the same instructions and 1–5 scale provided to the LLM. Both manual and LLM scores were grouped into three categories for agreement analysis: high (4–5), neutral (3), and low (1–2). The 5-point scale enables finer-grained analysis, while the 3-category grouping simplifies interpretation. Inter-rater agreement was assessed using Fleiss’ kappa. LLM accuracy was measured as the percentage of LLM trust-score assignments confirmed by human consensus (majority agreement among at least two of three reviewers). Inter-rater agreement among three human reviewers yielded Fleiss’ $\kappa$ = 0.685, indicating substantial agreement. Human consensus confirmed 82.2% of LLM trust-score assignments as correct. Agreement varied by authority: WHO (75.5% correct), CDC (82.0% correct), and FDA (89.0% correct). Raw validation results, including both 5-point manual ratings and LLM ratings, are provided in Supplementary Table S3.

### Trust score distributions

Following validation, trust score frequency distributions were tabulated by authority, with raw counts and percentages reported for each level. Ratio analyses assessed relative trust across HAs. High trust (4–5) to low trust (1–2) ratios were calculated for each authority to quantify the balance of positive versus negative expressions. Ratios of polarized expressions (1–2 and 4–5 combined) to neutral expressions (3) were computed to assess polarization versus neutrality for each authority.

### Mean trust score analysis

Trust scores were analyzed using descriptive statistics and one-way ANOVA to compare means across HAs. Post hoc comparisons used Tukey’s HSD test with family-wise error-rate correction to identify pairwise differences. Effect sizes were calculated using eta-squared ($\eta ^2$) to quantify observed differences.

### Trust dynamic analysis

Trust scores were aggregated by healthcare authority and quarter, yielding quarterly mean trust scores across all 17 quarters. Confidence intervals (95%) were calculated for each quarterly mean using the t-distribution to account for varying sample sizes. Visual analysis identified temporal patterns, convergence points, and authority rankings across the study period.

### Effect size analysis

To quantify changes in trust levels from the pre-pandemic baseline, we computed Cohen’s d by comparing 2019 trust levels with each subsequent quarter. 2019 was chosen as the baseline because it preceded COVID-19’s global impact. Trust scores were aggregated to daily means by healthcare authority, with days containing zero tweets excluded from calculations. Daily means from each quarter (Q1 2020–Q1 2023) were compared with the authority’s 2019 daily mean using Cohen’s d:

1 $$\begin{aligned} d = \frac{M_{\text {quarter}} - M_{2019}}{SD_{\text {pooled}}} \end{aligned}$$

where $M_{\text {quarter}}$ is the mean trust score for a given quarter, $M_{2019}$ is the 2019 baseline mean, and $SD_{\text {pooled}}$ is the pooled standard deviation. Effect sizes were interpreted using standard conventions: negligible ($|d| < 0.2$), small ($0.2 \le |d| < 0.5$), medium ($0.5 \le |d| < 0.8$), and large ($|d| \ge 0.8$).

### Volume-trust relationship analysis

To examine potential relationships between discussion volume and trust dynamics, we tested two hypotheses: volume-volatility association and volume-negativity association. Volume-volatility association tested whether increased discussion volume coincided with greater trust score variability within quarterly periods. Trust volatility was operationalized as the standard deviation of trust scores within each quarter, and we correlated quarterly standard deviations with quarterly tweet volumes for each authority. Volume-negativity association tested whether higher volume coincided with a greater proportion of low-trust expressions. For each authority, we correlated each quarter’s proportion of low-trust tweets (scores 1–2) with that quarter’s tweet volume. Statistical analysis used Spearman correlation coefficients.

### Topic modeling

**Fig. 1:**
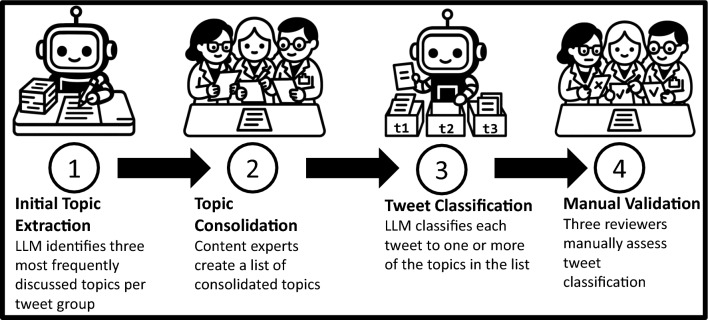
Topic-modeling methodology workflow. (1) Initial topic extraction using LLM analysis of quarterly tweet collections. (2) Expert consolidation into thematic categories. (3) Automated classification using consolidated frameworks. (4) Manual validation of classification accuracy by independent reviewers.

We conducted a four-stage topic-modeling process using Deepseek-r1 to identify key themes in healthcare professional discourse regarding healthcare authorities (Fig. 1). We divided the dataset into 102 groups based on healthcare authority, quarter, and trust level, then used Deepseek-r1 to identify the three most frequent topics in each group. The list of extracted topics is provided in Supplementary Table S2. Three content experts manually reviewed and consolidated the preliminary topics into comprehensive sets: 6 for low-trust tweets and 10 for high-trust tweets. The list of consolidated topics is provided in Supplementary Table S2. We then used Deepseek-r1 to classify individual tweets according to the consolidated topic lists. Topics appearing in fewer than 5% of analyzed tweets were consolidated into a single “others” category. Topic prevalence was calculated as the percentage of topic assignments within quarterly periods for each healthcare authority.

The topics emerged from data-driven analysis rather than from the OECD trust framework. Six of the ten major topics directly evaluate institutional attributes and correspond to the OECD framework’s two dimensions: integrity topics assess perceived values, competency topics assess perceived competencies, and directive topics assess policy outputs. The remaining four high-trust topics (vaccine positive messaging, medical innovation advances, global and environmental health, and health outreach activities) describe institutional activities rather than explicitly evaluating institutional attributes. Their consistent appearance in high-trust discourse suggests they may function as trust indicators, with HCPs who promote institutional outputs implicitly expressing confidence in the institution. These activity-focused topics may relate to competencies (vaccine messaging, medical innovation) or values (global health, outreach), though such mapping is interpretive and overlap between dimensions is possible.

To validate the LLM-based classification, three independent reviewers evaluated a stratified random sample of 300 tweets (100 per authority), with proportional representation of high-trust and low-trust groups within each authority. Reviewers assessed whether each LLM-assigned topic was correct and whether any important topics were missing. Inter-rater agreement was assessed using Fleiss’ kappa. LLM accuracy was measured as the percentage of LLM topic assignments confirmed by human consensus (majority agreement among at least two of three reviewers). Inter-rater agreement among reviewers yielded Fleiss’ $\kappa$ = 0.62, indicating substantial agreement. Human consensus confirmed 81.2% of LLM topic assignments as correct, with important topics missing in 6.8% of cases. Agreement was consistent across authorities: WHO (79.9% correct), CDC (82.3% correct), and FDA (81.3% correct). Raw results of topic-modeling validation are provided in Supplementary Table S3.

### Use of large language models

During the preparation of this work, the authors used Claude (Anthropic) and ChatGPT (OpenAI) to assist with grammar, wording, and phrasing; coding for statistical analyses and data visualization in Google Colab platform; and in LaTeX formatting. Additionally, Deepseek-r1 was used as described in “Trust score analysis” and “Topic modeling”. After using these tools, the authors reviewed and edited the content as needed and take full responsibility for the content of the published article.

## Results

### Tweet volume analysis

**Fig. 2:**
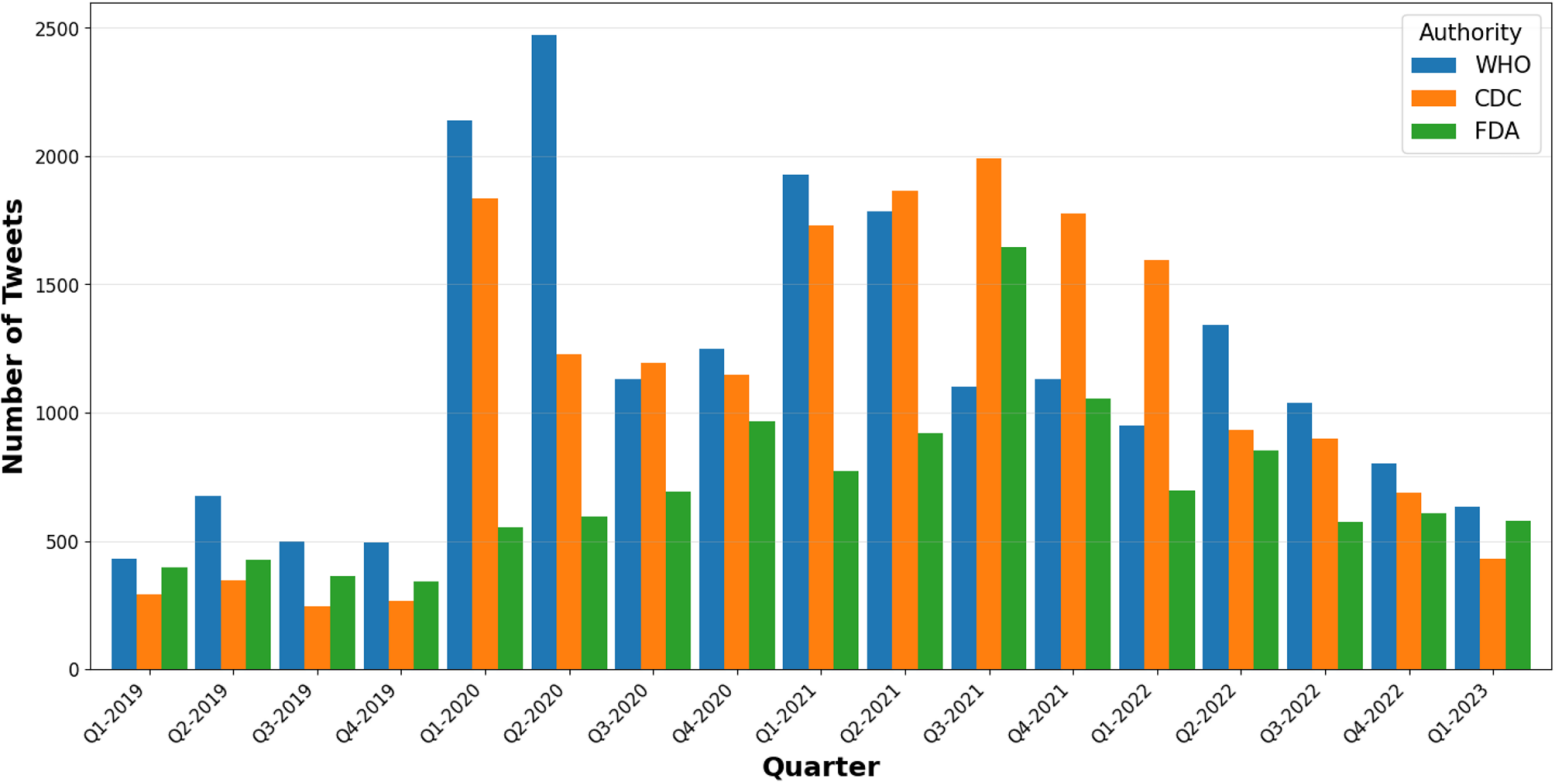
Tweet volume by healthcare authority per quarter from Q1 2019 through Q1 2023. Discourse volumes remained stable in 2019, increased sharply in early 2020, and peaked at different times across authorities.

In 2019, discourse volumes remained stable across authorities, with quarterly tweet counts ranging from 245 to 674. Tweet volumes increased from Q4 2019 to Q1 2020, with WHO rising 4.3-fold (493 to 2,139 tweets), CDC 6.9-fold (268 to 1,836), and FDA 1.6-fold (342 to 552). Peak volumes occurred at different times: WHO reached its maximum of 2,474 tweets in Q2 2020 (the highest single-quarter volume across all authorities), while CDC (1,992) and FDA (1,647) both peaked in Q3 2021. Through Q1 2023, all authorities showed declining trends from their peaks but remained above 2019 baseline levels, with Q1 2023 volumes of 634 (WHO), 431 (CDC), and 580 (FDA) (Figure 2).

### Trust score analysis

#### Trust score distributions

High-trust (scores 4–5) to low-trust (scores 1–2) ratios were highest for WHO (1.54), followed by FDA (0.89), and CDC (0.83). Ratios of polarized expressions (scores 1–2 and 4–5) to neutral expressions (score 3) were highest for CDC (1.18), followed by WHO (1.01), and FDA (0.81). Trust-score distributions are presented in Table 1.

**Table 1:** Trust score distribution by healthcare authority.

Authority	Trust score				
	1	2	3	4	5
WHO (*n* = 19,801)	820	3,093	9,866	5,911	111
	(4.1%)	(15.6%)	(49.8%)	(29.9%)	(0.6%)
CDC (*n* = 18,461)	1,397	4,070	8,453	4,448	93
	(7.6%)	(22.0%)	(45.8%)	(24.1%)	(0.5%)
FDA (*n* = 12,046)	428	2,432	6,644	2,497	45
	(3.5%)	(20.2%)	(55.2%)	(20.7%)	(0.4%)

#### Mean trust score analysis

Mean trust scores differed across authorities: WHO (M = 3.071, SD = 0.799), FDA (M = 2.942, SD = 0.750), and CDC (M = 2.879, SD = 0.877). The overall dataset mean was 2.970 (SD = 0.822) across 50,308 tweets. One-way ANOVA indicated significant differences, F(2, 50305) = 271.263, P < .001, with small effect size ($\eta ^2$ = 0.011). Tukey’s HSD post hoc comparisons revealed significant pairwise differences between all HAs: CDC had significantly lower scores than WHO (mean difference = 0.192, P < .001) and FDA (0.063, P < .001), while FDA had significantly lower scores than WHO (0.129, P < .001).

#### Trust dynamics analysis

**Fig. 3:**
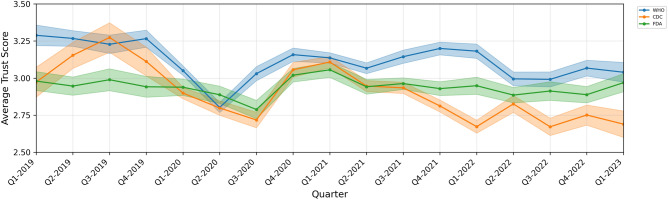
Trust dynamics across healthcare authorities from Q1 2019 through Q1 2023. Lines represent quarterly mean trust scores with 95% confidence intervals. FDA demonstrated the greatest stability, while CDC showed sustained low trust from Q4 2021 onward.

Trust trajectories across the three authorities exhibited distinct patterns (Fig. 3). FDA demonstrated the greatest stability with 0.27-point variability (range: 2.79–3.06), compared to WHO’s 0.49-point (range: 2.80–3.29) and CDC’s 0.60-point (range: 2.67–3.27). Two notable convergence points occurred: Q2 2020 with trust scores of 2.80 (WHO), 2.80 (CDC), and 2.89 (FDA), a 0.09-point spread; and Q1 2021 with scores of 3.14 (WHO), 3.11 (CDC), and 3.06 (FDA) yielding a 0.08-point spread. Both convergences were followed by divergences, with spreads expanding to 0.31 points in Q3 2020 and 0.39 points in Q4 2021. CDC’s relative ranking shifted markedly, consistently lowest from Q4 2021 through Q1 2023. CDC recorded the lowest trust scores of any authority, 2.67 in both Q1 2022 and Q3 2022, maintaining sustained low trust throughout 2022 and never exceeding 2.83 during this period. Tabulated quarterly trust scores by authority are provided in Supplementary Table S4.

#### Effect size analysis

Cohen’s d analysis revealed distinct patterns across authorities (Table 2). FDA maintained consistently small effect sizes across quarters, with most values below 0.20 in absolute terms, except Q3 2020 (d = −0.33) and Q3 2022 (d = −0.20). WHO showed the largest single effect size (d = −1.08 in Q2 2020), with large negative effects in Q2 2020, medium effects in Q1 2020 and Q3 2020, smaller effects through Q1 2022, and medium effects again from Q2 2022 through Q1 2023. CDC showed sustained medium negative effect sizes during 2022–2023, ranging from −0.50 to −0.79.

**Table 2:** Cohen’s d effect sizes for trust score changes from 2019 baseline by healthcare authority and quarter. Negative values indicate lower trust compared to 2019 baseline. Effect size interpretations: $|d| < 0.2$ = negligible, $0.2 \le |d| < 0.5$ = small, $0.5 \le |d| < 0.8$ = medium, $|d| \ge 0.8$ = large.

	**2020**				**2021**				**2022**				**2023**
	Q1	Q2	Q3	Q4	Q1	Q2	Q3	Q4	Q1	Q2	Q3	Q4	Q1
WHO	−0.54	−1.08	−0.54	−0.34	−0.33	−0.46	−0.31	−0.14	−0.19	−0.72	−0.71	−0.56	−0.56
CDC	−0.29	−0.52	−0.61	−0.14	−0.04	−0.27	−0.34	−0.24	−0.79	−0.50	−0.67	−0.62	−0.78
FDA	−0.05	−0.01	−0.33	0.01	0.14	0.01	−0.09	−0.14	0.04	−0.15	−0.20	−0.10	0.15

#### Volume-trust relationship analysis

Volume-volatility correlations varied across authorities: FDA showed the strongest relationship (r = 0.71, P < .01), followed by WHO with moderate correlation (r = 0.54, P = .02), while CDC showed no association (r = 0.00, P = .99). Volume-negativity associations were significant only for WHO (r = 0.63, P = .01), with FDA approaching significance (r = 0.42, P = .09) and CDC showing no significant correlation (r = 0.22, P = .39). WHO was the only authority to reach statistical significance across both hypotheses, while CDC showed no significant relationships in either.

### Topic modeling

**Fig. 4:**
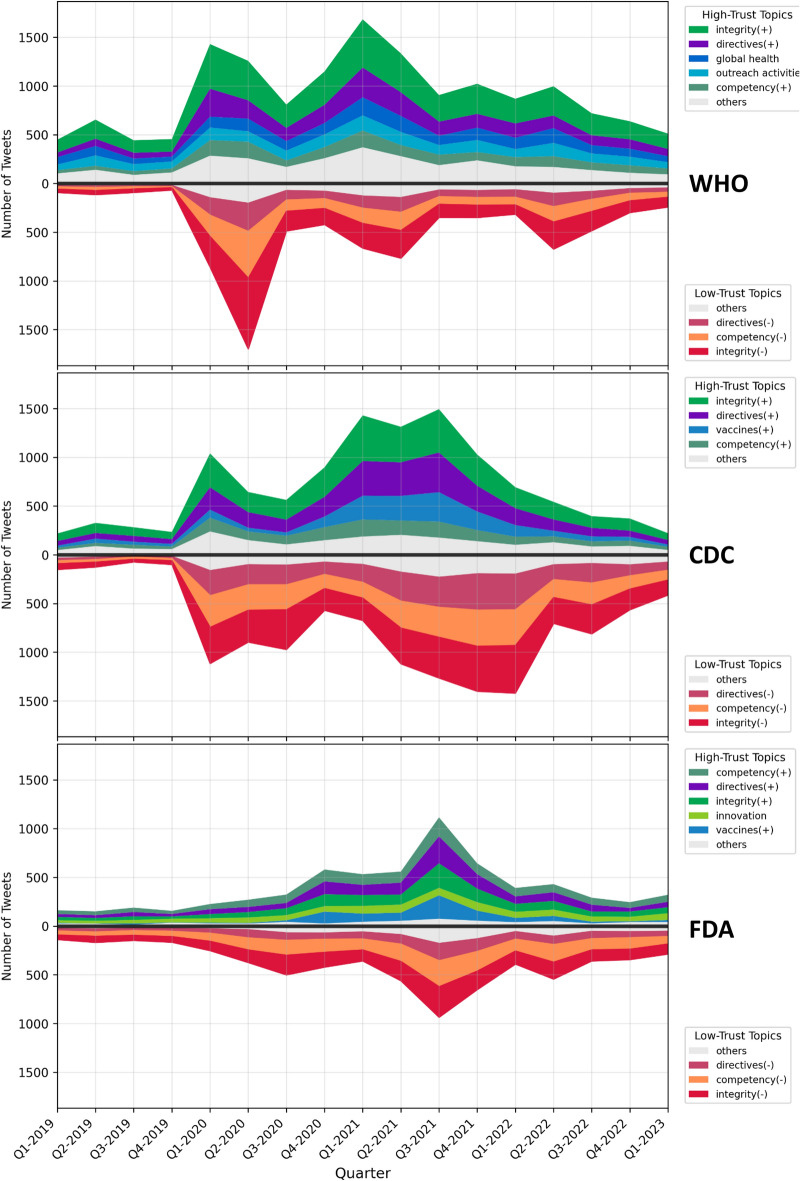
Topic distribution for WHO, CDC, and FDA from Q1 2019 through Q1 2023. Upper portions show high-trust topics; lower portions show low-trust topics. Integrity concerns dominated low-trust discourse across all authorities.

Following tweet classification, 10 of the 16 expert-identified topics emerged as major themes ($\ge$5% prevalence) across HAs. Table 3 presents the operational definitions and chart notations for these topics. Authority-specific distributions are shown in Fig. 4.

**Table 3:** Major topic operational definitions and chart notations.

Topic names	Operational definition	Notation
**Low trust**		
Institutional integrity concerns	Doubting the credibility, transparency, or independence of public-health institutions	integrity(−)
Institutional competency concerns	Criticizing the competence, procedures, or decision-making processes of HAs or regulators	competency(−)
Unhelpful health directives	Criticizing the content or impact of published health policies, guidelines, rules, or mandates	directives(−)
**High trust**		
Institutional integrity affirmation	Recognizing public-health institutions for their credibility, transparency, and ethical communication	integrity(+)
Institutional competency soundness	Praising rigorous, efficient, evidence-based decision-making processes of HAs or regulators	competency(+)
Beneficial health directives	Presenting or endorsing official health policies or clinical guidelines	directives(+)
Vaccine positive messaging	Promoting vaccination information related to disease prevention and public health benefits	vaccines(+)
Medical innovation advances	Announcing or describing newly approved medications, devices, or treatments with promising clinical applications	innovation
Global and environmental health	Highlighting progress and cooperation in global health or environmental sustainability efforts	global health
Health outreach activities	Showcasing professional events or initiatives that share medical knowledge or promote health awareness	outreach activities

Topic patterns showed both universal and authority-specific characteristics. Leading high-trust topics varied by authority: WHO and CDC led with institutional integrity affirmation (19.9% and 15.5% respectively), while FDA’s leading high-trust topic was beneficial health directives (10.6%). Low-trust topic rankings were identical across all authorities: integrity concerns ranked highest, followed by competency concerns, then unhelpful health directives.

Authority-unique topics distinguished discourse patterns: WHO emphasized global and environmental health (7.7%) and health-outreach activities (7.5%), while FDA emphasized medical-innovation advances (7.3%). Integrity and competency topics appeared in complementary positive-negative pairs across authorities. For integrity, WHO exhibited more positive than negative discourse (19.9% affirmation vs 13.5% concerns), while CDC and FDA showed the reverse: CDC (18.3% concerns vs 15.5% affirmation) and FDA (17.1% concerns vs 10.3% affirmation). For competency, all authorities showed more negative than positive discourse: WHO (8.6% concerns vs 6.7% soundness), CDC (13.4% concerns vs 6.1% soundness), and FDA (15.7% concerns vs 10.6% soundness). Complete topic prevalence data across all authorities are provided in Supplementary Table S5.

## Discussion

This study analyzed 50,308 tweets from 9,442 HCPs mentioning WHO, CDC, and FDA between January 2019 and March 2023. Trust levels toward these authorities were assessed, and topic modeling was performed to identify discourse themes. Trust patterns were compared across authorities to examine institutional trust dynamics during COVID-19.

Effect size analysis revealed that FDA experienced minimal trust decline from baseline (negligible effect sizes), while CDC and WHO showed larger negative effects with greater volatility across time. WHO experienced the largest single-quarter decline (Cohen’s d = −1.08 in Q2 2020), while CDC showed sustained medium-to-large negative effects throughout 2022–2023. Topic modeling identified 10 major themes across authorities. Across all authorities, integrity concerns dominated low-trust discourse more than competency concerns, while integrity affirmation led high-trust discourse. Authority-specific patterns emerged: WHO emphasized global health themes, FDA emphasized medical innovation, and CDC showed the highest polarization ratio.

These trust patterns may be interpreted in the context of institutional events during the study period, though attributing trends to individual events is necessarily an oversimplification, as trust reflects multiple overlapping factors^17^. The following three cases illustrate trust changes that coincided with specific institutional events. First, the Q2 2020 trust decline for WHO and CDC coincided with criticism of WHO’s early pandemic response, including perceived delays in early warnings and precautionary measures^26^, and CDC’s diagnostic testing difficulties that limited the nation’s detection capacity^27^; FDA showed negligible trust change during this period (Cohen’s d = −0.01). Second, in Q1 2021, trust levels approached pre-pandemic values, in temporal proximity to the FDA’s emergency use authorizations for the Pfizer-BioNTech (December 11, 2020) and Moderna (December 18, 2020) COVID-19 vaccines and the initial phase of vaccine deployment. Third, the sustained erosion of CDC trust beginning in late 2021 occurred after a series of guidance reversals, including the reinstatement of indoor mask recommendations for vaccinated individuals in July 2021 (reversing guidance issued two months earlier) and the shortened COVID-19 isolation period from ten to five days announced in December 2021; a survey of US adults conducted shortly after the isolation policy change found that 26% reported lower trust in CDC recommendations and 44% perceived that economic factors influenced CDC guidance^28^. These temporal associations do not establish causal relationships between specific events and trust changes.

These results align with prior longitudinal studies of the general population: a global study of 44,775 participants across 40 countries (2017–2020) found declining trust in WHO from pre-pandemic to pandemic periods^29^; and a study of approximately 750 US adults (2020–2024) found sharper declines in CDC trust than in FDA trust^30^. These patterns suggest that CDC and WHO may be more vulnerable to trust erosion than FDA during health crises.

Two possible contributing factors may explain differences in trust vulnerability between HAs: politicization and institutional mandate. First, perceived politicization may contribute to trust erosion. Given evidence that politicization undermines institutional trust ^31,32^, it is reasonable to expect that differing trust patterns reflect differing perceived levels of politicization across authorities. However, while studies document that WHO ^33^, CDC ^34^, and FDA ^35^ were all perceived as political during COVID-19, no research compares their relative politicization among healthcare professionals. Because comparative politicization data are lacking, this potential factor cannot be empirically evaluated. Second, the breadth of institutional mandate may contribute to the observed patterns. CDC and WHO operate across broad domains affecting both professional practice and personal life, whereas FDA’s narrower mandate focuses on drug and device approvals. Authorities with broader mandates may face greater scrutiny across domains, creating more opportunities for conflicts that erode trust in professional autonomy and clinical judgment. However, no research directly examines the relationship between institutional mandate breadth and trust vulnerability among healthcare professionals, though this remains a plausible explanatory mechanism.

The consistent prioritization of integrity over competency in HCPs’ discourse aligns with the OECD framework’s two drivers of institutional trust: values (integrity) and competencies ^5^. While the OECD framework does not specify relative weighting, our finding that integrity concerns dominated low-trust discourse suggests that perceived violations of values may be more damaging to trust than perceived failures of competence during large-scale health crises. This interpretation aligns with evidence that honesty and integrity weigh more heavily for trust than competence ^36–38^.

The following limitations should be considered when interpreting our findings. Twitter users represent a non-random sample of HCPs, with US platform demographics skewing younger, more educated, and more politically liberal than the general population, each of which may introduce biases in trust expressions ^39^. Platform engagement further concentrates influence; the top 10% of users generate 92% of content, and these prolific users lean Democratic (69% Democratic versus 26% Republican) ^40^. Given evidence that political liberals show higher trust in CDC and WHO ^33^, these demographics may amplify some trust perspectives while underrepresenting others.

LLM-based classifications are imperfect. Our validations in “Validation of trust score analysis” and “Topic modeling” indicate that individual tweet-level classifications are accurate in 80–85% of cases. Therefore, results should be interpreted as reflecting aggregate patterns rather than precise individual assessments. Additionally, trust expressions on public platforms may reflect performative aspects of professional identity rather than private beliefs. These findings suggest two categories of practical application: monitoring trust and building trust through targeted responses. Early detection of trust erosion can enable timely corrective measures, mitigating potential damage^41^. The feasibility of quantifying trust through social media creates opportunities for real-time monitoring, as such analysis has proven effective for tracking population health and crisis responses^42^. HAs could use social media-based monitoring to detect early signals of trust erosion, enabling proactive responses at short notice.

Building trust may require approaches informed by identified discourse patterns. Because integrity concerns consistently outweighed competency concerns across authorities, efforts to prevent distrust may be more effective if they prioritize integrity over competency. Integrity-focused approaches could include greater transparency in decision-making and clear disclosure of potential conflicts of interest^43,44^. Beyond shared findings, the distinct trust profiles of individual organizations highlight the need for tailored trust-building approaches, consistent with literature on context-specific trust strategies^45,46^. Each organization can use monitoring data to identify its specific trust-eroding topics and implement targeted responses.

The underexplored area of HCPs’ trust in HAs warrants further investigation through several directions. Behavioral studies could test whether social media trust expressions correlate with clinical practice or guideline adherence, validating digital discourse as a trust indicator. Longitudinal analyses of post-pandemic trust recovery could inform evidence-based strategies for rebuilding institutional credibility. Cross-platform studies could test whether patterns observed on Twitter generalize to other digital platforms. Finally, methodological research combining social media analysis with surveys could develop hybrid methods that leverage both real-time digital monitoring and structured measurement.

## Supplementary Information

Supplementary Information 1.

Supplementary Information 2.

Supplementary Information 3.

Supplementary Information 4.

Supplementary Information 5.

Supplementary Information 6.

Supplementary Information 7.

## Data Availability

All data supporting the findings of this study are available within the paper and its Supplementary Information. De-identified datasets containing trust score assignments and topic classifications are provided as Supplementary Data S2, including healthcare authority labels and dates but excluding tweet text and user identifiers to protect privacy and comply with Twitter’s terms of service. Analysis code is available as Supplementary Data S1.
