# Supplementary material for: Healthcare professionals’ trust in health authorities throughout COVID-19: a social media analysis
Source: Sci Rep. 2026 Apr 30;16:20082. doi: 10.1038/s41598-026-50565-4 (PMC13324178; doi:10.1038/s41598-026-50565-4)
Supplement: Supplementary file 3 — Supplementary Information 3. [file 41598_2026_50565_MOESM3_ESM.pdf]

Supplementary Information

Healthcare Professionals' Trust in Health Authorities Throughout COVID-19: A Social Media Analysis

Idan-Chaim Cohen[a,b,*], Noa Tal[b,c], Aviad Elyashar[b,d], Rami Puzis[b,c,†], Odeya Cohen[a,†]

[a] Department of Nursing, Recanati School of Community Health Professions, Faculty of Health Sciences, Ben-Gurion University of the Negev, Beer-Sheva, Israel

[b] Cyber@BGU, Ben-Gurion University of the Negev, Beer-Sheva, Israel

[c] Department of Software and Information Systems Engineering, Ben-Gurion University of the Negev, Beer-Sheva, Israel

[d] Department of Computer Science, Shamoon College of Engineering, Beer-Sheva, Israel

* Corresponding author: idanchai@bgu.ac.il

† These authors contributed equally to this work (shared supervision)

Supplementary Note: STROBE Checklist

STROBE Statement—Checklist of Items for Observational Studies

Section/Topic	Item No	Recommendation	Section
Title and abstract	1	(a) Indicate the study's design with a commonly used term in the title or the abstract (b) Provide in the abstract an informative and balanced summary of what was done and what was found	Title, Abstract
**Introduction**			
Background/rationale	2	Explain the scientific background and rationale for the investigation being reported	1.1-1.4
Objectives	3	State specific objectives, including any prespecified hypotheses	1.5
**Methods**			
Study design	4	Present key elements of study design early in the paper	Abstract, 2.2-2.5
Setting	5	Describe the setting, locations, and relevant dates, including periods of recruitment, exposure, follow-up, and data collection	2.2, Abstract
Participants	6	(a) Cohort study—Give the eligibility criteria, and the sources and methods of selection of participants. Describe methods of follow-up Case-control study—Give the eligibility criteria, and the sources and methods of case ascertainment and control selection. Give the rationale for the choice of cases and controls Cross-sectional study—Give the eligibility criteria, and the sources and methods of selection of participants	2.2
		(b) Cohort study—For matched studies, give matching criteria and number of exposed and unexposed Case-control study—For matched studies, give matching criteria and the number of controls per case	N/A
Variables	7	Clearly define all outcomes, exposures, predictors, potential confounders, and effect modifiers. Give diagnostic criteria, if applicable	2.3, 2.4, 2.5
Data sources/ measurement	8*	For each variable of interest, give sources of data and details of methods of assessment (measurement). Describe comparability of assessment methods if there is more than one group	2.2, 2.4, 2.5
Bias	9	Describe any efforts to address potential sources of bias	2.4.1, 4.4
Study size	10	Explain how the study size was arrived at	2.2
Quantitative variables	11	Explain how quantitative variables were handled in the analyses. If applicable, describe which groupings were chosen and why	2.3, 2.4
Statistical methods	12	(a) Describe all statistical methods, including those used to control for confounding	2.4.3-2.4.6
		(b) Describe any methods used to examine subgroups and interactions	2.4.6
		(c) Explain how missing data were addressed	N/A
		(d) Cohort study—If applicable, explain how loss to follow-up was addressed Case-control study—If applicable, explain how matching of cases and controls was addressed Cross-sectional study—If applicable, describe analytical methods taking account of sampling strategy	N/A
		(e) Describe any sensitivity analyses	N/A
**Results**			
Participants	13*	(a) Report numbers of individuals at each stage of study—eg numbers potentially eligible, examined for eligibility, confirmed eligible, included in the study, completing follow-up, and analysed	3.1, Abstract

Section/Topic	Item No	Recommendation	Section
		(b) Give reasons for non-participation at each stage	N/A
		(c) Consider use of a flow diagram	N/A
Descriptive data	14*	(a) Give characteristics of study participants (eg demographic, clinical, social) and information on exposures and potential confounders	3.1, 3.2.2
		(b) Indicate number of participants with missing data for each variable of interest	N/A
		(c) Cohort study—Summarise follow-up time (eg, average and total amount)	N/A
Outcome data	15*	Cohort study—Report numbers of outcome events or summary measures over time Case-control study—Report numbers in each exposure category, or summary measures of exposure Cross-sectional study—Report numbers of outcome events or summary measures	3.2
Main results	16	(a) Give unadjusted estimates and, if applicable, confounder-adjusted estimates and their precision (eg, 95% confidence interval). Make clear which confounders were adjusted for and why they were included	3.2.3-3.2.5
		(b) Report category boundaries when continuous variables were categorized	N/A
		(c) If relevant, consider translating estimates of relative risk into absolute risk for a meaningful time period	N/A
Other analyses	17	Report other analyses done—eg analyses of subgroups and interactions, and sensitivity analyses	3.2.6, 3.2.7
**Discussion**			
Key results	18	Summarise key results with reference to study objectives	4
Limitations	19	Discuss limitations of the study, taking into account sources of potential bias or imprecision. Discuss both direction and magnitude of any potential bias	4.4
Interpretation	20	Give a cautious overall interpretation of results considering objectives, limitations, multiplicity of analyses, results from similar studies, and other relevant evidence	4.1-4.3
Generalisability	21	Discuss the generalisability (external validity) of the study results	4.4
**Other information**			
Funding	22	Give the source of funding and the role of the funders for the present study and, if applicable, for the original study on which the present article is based	N/A (omitted for blinded review)

**Give information separately for cases and controls in case-control studies and, if applicable, for exposed and unexposed groups in cohort and cross-sectional studies.*

Note: An Explanation and Elaboration article discusses each checklist item and gives methodological background and published examples of transparent reporting. Information on the STROBE Initiative is available at www.strobe-statement.org.

Supplementary Methods: LLM Prompts

This section presents the complete prompts used for the large language model analysis in our study.

Prompt 1: Trust Level Analysis

"You will be given a tweet mentioning {HCA}. Evaluate the tweet author's level of trust in {HCA} using the following scale: 1 - No trust 2 - Low trust 3 - Neutral or unclear trust 4 - High trust 5 - Complete trust Provide only the corresponding number (1, 2, 3, 4, or 5) as your response, without any additional text or characters. Tweet Text: {tweet text}"

Comment: In this prompt template, the placeholder {HCA} was systematically replaced with the specific authority name (WHO, CDC, or FDA) relevant to each tweet, while {tweet text} was populated with the verbatim content of the tweet under analysis.

Prompt 2: Initial Topic Extraction

"I will provide you with tweets that mention {HCA}. Analyze these tweets and determine the three most frequently discussed topics. Then, reply with exactly three lines in the exact format below (no additional text, commentary, or formatting is allowed): 1: {topic 1} 2: {topic 2} 3: {topic 3} It is critical that you adhere to the formatting instructions. Output only the three lines as instructed. Here are the tweets: {tweet text} Remember - Only three lines with topics with no additional text."

Comment: In this prompt template, the placeholder {HCA} was replaced with the specific authority name (WHO, CDC, or FDA), and {tweet text} was populated with the collection of tweets from the respective quarterly period for that authority, filtered by trust level (distrust or trust).

Prompt 3: Classification of Trust-Expressing Tweets

"You will receive the text of one tweet. Task: Identify every subject the tweet raises from the list below and return only a Python list (["...", ...]).

Subjects:

1. Vaccine Positive Messaging – promoting vaccination information related to disease prevention and public health benefits
2. Beneficial Health Directives – presenting or endorsing official health policies or clinical guidelines
3. Institutional Integrity Affirmation – recognizing public-health institutions for their credibility, transparency, and ethical communication
4. Institutional Competency Soundness – praising rigorous, efficient, evidence-based decision-making processes of health authorities or regulators
5. Healthcare Safety Resource Improvements – highlighting adequate PPE, testing capacity, or safety protocols that protect healthcare staff
6. Mental Health Support Successes – demonstrating effective psychological-support systems or mental-health initiatives
7. Global and Environmental Health – highlighting progress and cooperation in global health or environmental sustainability efforts
8. Medical Innovation Advances – announcing or describing newly approved medications, devices, or treatments with promising clinical applications
9. Health Outreach Activities – showcasing professional events or initiatives that share medical knowledge or promote health awareness

10. Healthcare Workforce Development – discussing initiatives, investments, or collaborations that strengthen, support, or recognize HCPs

Output Rules:

• Return a Python list containing at most three subject names.
• List each applicable subject exactly as written above. If more than three subjects apply, include the three most salient.
• If the tweet mentions another health-related subject not covered by 1–10, append one item in the form "other: SHORT_TOPIC" (≤ 4 words) as part of the three allowed items.
• No duplicates, explanations, or extra text - return the list and nothing else. Input format supplied to you: Text of a tweet: "{TWEET TEXT HERE}""

Comment: In this prompt template, the placeholder {TWEET TEXT HERE} was replaced with the verbatim content of individual tweets that had been assigned trust scores of 4 or 5 in the previous analysis step.

Prompt 4: Classification of Distrust-Expressing Tweets

"You will receive the text of one tweet. Task: Identify every subject the tweet raises from the list below and return only a Python list (["...", ...]).

Subjects:

1. Vaccine Controversy – questioning vaccine safety, efficacy, or ingredients
2. Unhelpful Health Directives – criticizing the content or impact of published health policies, guidelines, rules, or mandates
3. Institutional Integrity Concerns – doubting the credibility, transparency, or independence of public-health institutions
4. Institutional Competency Concerns – criticizing the competence, procedures, or decision-making processes of health authorities or regulators
5. Healthcare Safety Resource Issues – shortages or inadequacies in PPE, tests, or safety protocols that protect healthcare staff
6. Mental Health Concerns – highlighting inadequate psychological-support systems or risk factors

Output Rules:

• Return a Python list containing at most three subject names.
• List each applicable subject exactly as written above. If more than three subjects apply, include the three most salient.
• If the tweet mentions another health-related subject not covered by 1–6, append one item in the form "other: SHORT_TOPIC" (≤ 4 words) as part of the three allowed items.
• No duplicates, explanations, or extra text - return the list and nothing else. Input format supplied to you: Text of a tweet: "{TWEET TEXT HERE}""

Comment: In this prompt template, the placeholder {TWEET TEXT HERE} was replaced with the verbatim content of individual tweets that had been assigned trust scores of 1 or 2 in the previous analysis step.

Supplementary Table S1: Regular Expression Search Terms

Regex search terms used to identify tweets mentioning health authorities.

Health Authority	Search Terms
WHO	WHO<br>World Health Organization<br>@WHO
CDC	CDC<br>Centers for Disease Control and Prevention<br>@CDCgov
FDA	FDA<br>US FDA<br>Food and Drug Administration<br>United States Food and Drug Administration<br>@US_FDA

Note: To distinguish cases in which the term WHO was referring to the World Health Organization from those in which the term is used as a pronoun (e.g., in sentences like "Who let the dogs out?"), we exclusively searched for the uppercase form "WHO", thereby excluding irrelevant instances of "who" or "Who".

Supplementary Table S6. Synthetic Illustrative Examples of Trust Levels

Synthetic examples created for illustrative purposes only. These are not actual tweets from the dataset. Examples are designed to reflect the range of trust expressions observed across healthcare authorities.

Trust Score	Label	Synthetic Illustrative Example
1	No trust	The @US_FDA is lying to the public about effective treatments. They have zero credibility left. How many patients have to suffer before they stop putting politics over science?? #HealthcareFailure
2	Low trust	Still struggling to see how the data justifies @WHO's recommendation here. The evidence is weak and the approval process seemed rushed. We need more rigorous standards. #EvidenceMatters
3	Neutral or unclear	Big news from the @CDCgov today on respiratory illness protocols. Will review the details before our next staff meeting. https://t.co/example
4	High trust	Drug safety requires @US_FDA oversight. If we want to call something medicine, it should go through proper research, testing, and approval like everything else. #PatientSafety
5	Complete trust	My confidence in the @CDCgov has grown enormously watching their team work through this. Transparent, evidence-based, exactly what public health leadership should look like. #PublicHealth
