# Supplementary material for: Healthcare professionals’ trust in health authorities throughout COVID-19: a social media analysis
Source: Sci Rep. 2026 Apr 30;16:20082. doi: 10.1038/s41598-026-50565-4 (PMC13324178; doi:10.1038/s41598-026-50565-4)
Supplement: Supplementary file 7 — Supplementary Information 7. [file 41598_2026_50565_MOESM7_ESM.pdf]

Complete Topic Prevalence by Healthcare Authority

Topic	WHO (%)	CDC (%)	FDA (%)
Low Trust Topics			
Institutional Integrity Concerns	13.5	18.3	17.1
Institutional Competency Concerns	8.6	13.4	15.7
Unhelpful Health Directives	6.4	12.3	9.2
Vaccine Controversy	1.3	3.3	4.4
Healthcare Safety Resource Issues	0.9	1.6	0.6
Mental Health Concerns	0.5	0.6	0.4
High Trust Topics			
Institutional Integrity Affirmation	19.9	15.5	10.3
Beneficial Health Directives	10.4	11.7	10.6
Institutional Competency Soundness	6.7	6.1	10.6
Global and Environmental Health	7.7	0.6	0.1
Health Outreach Activities	7.5	3.0	1.9
Medical Innovation Advances	1.2	0.4	7.3
Vaccine Positive Messaging	4.3	6.9	6.3
Healthcare Workforce Development	2.9	0.9	0.7
Healthcare Safety Resource Improvements	2.1	1.6	1.0
Mental Health Support Successes	1.0	0.5	0.3
Other Topics			
Others[a]	5.0	3.4	3.4

Note: "Others" represents consolidated topics with prevalence below 5% threshold that were grouped for visualization purposes.
